# Predicting the risk of pancreatic cancer in adults with new-onset diabetes: development and internal–external validation of a clinical risk prediction model

**DOI:** 10.1038/s41416-024-02693-9

**Published:** 2024-05-03

**Authors:** Ash Kieran Clift, Pui San Tan, Martina Patone, Weiqi Liao, Carol Coupland, Rachael Bashford-Rogers, Shivan Sivakumar, Julia Hippisley-Cox

**Affiliations:** 1 Nuffield Department of Primary Care Health Sciences, University of Oxford, Oxford, UK; 2 Cancer Research UK Oxford Centre, University of Oxford, Oxford, UK; 3 Centre for Academic Primary Care, School of Medicine, University of Nottingham, Nottingham, UK; 4 Wellcome Centre for Human Genetics, University of Oxford, Oxford, UK; 5 Department of Biochemistry, University of Oxford, Oxford, UK; 6 Institute of Immunology and Immunotherapy, Birmingham Medical School, Birmingham, UK; 7 Cancer Centre, Queen Elizabeth Hospital, University Hospitals of Birmingham NHS Trust, Birmingham, UK

**Keywords:** Epidemiology, Risk factors

## Abstract

**Background:**

The National Institute for Health and Care Excellence (NICE) recommends that people aged 60+ years with newly diagnosed diabetes and weight loss undergo abdominal imaging to assess for pancreatic cancer. More nuanced stratification could lead to enrichment of these referral pathways.

**Methods:**

Population-based cohort study of adults aged 30–85 years at type 2 diabetes diagnosis (2010–2021) using the QResearch primary care database in England linked to secondary care data, the national cancer registry and mortality registers. Clinical prediction models were developed to estimate risks of pancreatic cancer diagnosis within 2 years and evaluated using internal–external cross-validation.

**Results:**

Seven hundred and sixty-seven of 253,766 individuals were diagnosed with pancreatic cancer within 2 years. Models included age, sex, BMI, prior venous thromboembolism, digoxin prescription, HbA1c, ALT, creatinine, haemoglobin, platelet count; and the presence of abdominal pain, weight loss, jaundice, heartburn, indigestion or nausea (previous 6 months). The Cox model had the highest discrimination (Harrell’s *C*-index 0.802 (95% CI: 0.797–0.817)), the highest clinical utility, and was well calibrated. The model’s highest 1% of predicted risks captured 12.51% of pancreatic cancer cases. NICE guidance had 3.95% sensitivity.

**Discussion:**

A new prediction model could have clinical utility in identifying individuals with recent onset diabetes suitable for fast-track abdominal imaging.

## Introduction

Pancreatic cancer has a very poor prognosis, with less than a quarter of patients surviving past one year of diagnosis [1]. Most patients are diagnosed with advanced disease. Improved pancreatic cancer outcomes could be attainable with earlier detection, but in the absence of a screening programme this is challenged by minimal or vague presenting symptoms in early-stage disease, mandating the exploration of alternative approaches.

One approach could target the association between type 2 diabetes mellitus (T2DM) and pancreatic cancer—up to 1% of adults with new-onset T2DM develop pancreatic cancer within 3 years [2], and 1 in 4 pancreatic cancer patients have diabetes [3]. Although this association’s directionality and mechanisms are incompletely understood [4, 5], people with new-onset diabetes represent an important opportunity for identifying high-risk sub-populations suitable for further testing—new-onset ‘T2DM’ may in fact in some cases be type 3c pancreatogenic diabetes caused by an underlying pancreatic cancer.

Current guidance in the United Kingdom from the National Institute for Health and Care Excellence (NICE) recommends 2-week-wait abdominal imaging for people aged over 60 years with new-onset T2DM and weight loss [6]. However, integrating additional factors in the form of multivariable prediction models could provide a more nuanced estimation of individual risks to enrich referral pathways. These could include pre-existing conditions, symptoms, or blood markers routinely measured in primary care [7]. A recent population-based case–control study using primary care data from England (over 28,000 individuals with pancreatic ductal adenocarcinoma (PDAC)) reported significantly increased odds of pancreatic cancer in those with recorded comorbidities including acute pancreatitis and inflammatory bowel disease, and that results from commonly used blood tests or body mass index (BMI) may have detectable changes or trends up to 3 years prior to cancer diagnosis [7]. Related work using primary care data has also shown that the symptom profiles for PDAC and pancreatic neuroendocrine neoplasms (PNEN) overlap, with both showing an association between T2DM and increased risk [3].

Several groups have developed clinical prediction models that estimate the risk of pancreatic cancer diagnosis in people with recent-onset T2DM. These vary in sample size and analytical approach, and some have significant limitations. The END-PAC score that integrates age, change in weight and change in blood glucose was developed and evaluated in a very small sample size (64 cancer cases in development, only 9 in validation) [8]. The study of Boursi and colleagues developed a prediction model using data from 109,385 individuals with new-onset T2DM in The Health Improvement Network (THIN) database, of which 390 were diagnosed with pancreatic cancer within 3 years. This study reported a final area under the curve (AUC) of 0.82 (95% confidence interval (CI): 0.75–0.89), but used logistic regression to handle time-to-event data and did not assess model calibration. This is rarely assessed in existing models, as is clinical utility [9], rendering the potential usefulness of these tools uncertain [10]. Furthermore, there is interest in the potential scope for machine learning in clinical prediction, but the relative performance and incremental yield of these flexible techniques compared to regression methods has varied across reports in low-dimensional clinical settings [11].

This study aimed to use large-scale, population-representative, linked electronic health record datasets to develop and robustly evaluate clinical prediction models to estimate individual-level risks of developing pancreatic cancer within 2 years of T2DM diagnosis. Using the QResearch database, this study is the largest of its kind, leverages national dataset linkages to improve outcome ascertainment, and considers the broadest range of clinically relevant symptoms, comorbidities and measurements yet. The study compares three modelling approaches and compares the results against the current referral criteria recommended by NICE.

## Methods

We undertook an open cohort study. We compared three modelling strategies to predict the 2-year risk of pancreatic cancer diagnosis following a new diagnosis of T2DM in people aged 30–85 years: Cox proportional hazards modelling, and two machine learning approaches (XGBoost and artificial neural networks). The protocol is available elsewhere [12] and this study is reported in accordance with TRIPOD guidelines [13]. As discussed above, in some cases T2DM may, in actuality, be type 3c diabetes, but as the initial diagnosis made in primary care is likely to be considered and recorded as T2DM, this case definition is used. The prediction horizon of 2 years was deemed clinically meaningful by the study group and aligns with previous prediction model development and validation research using the QResearch database [14]. The prediction horizon and the age range of the model’s target population were agreed with the funder (Pancreatic Cancer UK) and patient and public involvement panel members. Further, the age range of the target population is similar to that of previous studies (e.g. those aged 35 and older) [15] and recognises the increasing incidence of diabetes mellitus in adults before middle age.

### Data sources and study population

The QResearch database (version 46) was used, which has collected anonymised, routine clinical data from over 1500 general practices in the United Kingdom including diagnoses, clinical measurements and prescriptions. This is linked at the individual level to NHS Digital’s Hospital Episodes Statistics (HES), the national cancer registry and the Office for National Statistics’ mortality register.

Adults aged 30–85 years registered with a general practice contributing data to the QResearch database between 1 January 2010 and 1 July 2021 were identified. Patients were eligible from the latest date of: their 30th birthday (since diagnoses under this age are extremely rare), the date on which their general practice had contributed data to QResearch for at least 1 year, or the date on which they had been registered with their practice for 1 year.

Using SNOMED codes, a cohort of individuals that received a new diagnosis of ‘T2DM’ in primary care were identified. Those with previous recorded diagnoses of pancreatic cancer on GP, HES, or cancer registry records were excluded. Those that had recorded prescriptions for anti-diabetes medications prior to their recorded date of T2DM were excluded. Cohort derivation is summarised in Supplementary Fig. 1.

### Outcomes and candidate predictors

The outcome of interest was diagnosis of pancreatic cancer of any histological type (e.g. ductal adenocarcinoma, neuroendocrine neoplasm), recorded on any of the linked datasets. Follow-up was calculated from the prediction date (i.e. the intended point of model use, which is the date of T2DM diagnosis plus 2 weeks) until date of pancreatic cancer diagnosis or censoring (reached 2 years without event, left practice, or died from another event). This prediction date of 2 weeks from diabetes diagnosis was chosen to align with the clinical scenario where general practitioners may request additional blood tests once a diagnosis has been made. Blood test results were considered as candidate predictors—this permitted use of most recent data available at the point of the model’s intended use during model development and validation. Candidate predictors were identified from review of clinical and epidemiological literature [12], see Table 1. Clinical codes used in this study are available at https://www.qresearch.org/qcode-group-library/.

**Table 1:** Characteristics of the study cohort.

Parameter	Category	Pancreatic cancer diagnosed (Col %)	Pancreatic cancer not diagnosed (Col %)
Total	Number	767	252,999
Age at diagnosis of type 2 diabetes	Mean (SD)	70.1 (9.1)	60.1 (12.5)
Sex	Female	339 (44.2)	108,558 (42.8)
	Male	428 (55.8)	145,208 (57.2)
Townsend deprivation score fifth	1 (most affluent)	234 (30.51)	56,942 (22.51)
	2	210 (27.38)	53,423 (21.12)
	3	134 (17.47)	52,495 (20.75)
	4	122 (15.91)	47,308 (18.70)
	5 (most deprived)	67 (8.74)	42,263 (16.70)
	Not recorded	0	568 (0.22)
Ethnicity	White	582 (75.9)	166,675 (65.7)
	South Asian	20 (2.6)	27,312 (10.8)
	Black	17 (2.2)	12,801 (5.0)
	Other	20 (2.6)	14,941 (5.9)
	Not recorded	128 (16.7)	32,027 (12.6)
Smoking status	Non-smoker	353 (46.0)	126,840 (50.0)
	Ex-smoker	270 (35.2)	81,121 (32.0)
	Light smoker (1–9/day)	80 (10.4)	22,327 (8.8)
	Moderate smoker (10–19/day)	39 (5.1)	12,846 (5.1)
	Heavy smoker (20+/day)	22 (2.9)	10,117 (4.0)
	Not recorded	<10	515 (0.2)
Alcohol intake	Non-drinker	302 (39.4)	112,447 (44.3)
	Trivial <1 u/day	195 (25.4)	64,131 (25.3)
	Light 1–2 u/day	114 (14.9)	28,223 (11.1)
	Moderate 3–6 u/day	108 (14.1)	30,108 (11.9)
	Heavy or very heavy 7+ u/day	17 (2.2)	5465 (2.2)
	Not recorded	31 (4.0)	13,392 (5.3)
BMI	Not recorded	125 (16.3)	42,482 (16.7)
	Mean (SD)	29.5 (5.7)	32.7 (6.7)
Acute pancreatitis		22 (2.9)	3092 (1.2)
Chronic pancreatitis		14 (1.8)	1695 (0.7)
Venous thromboembolism		52 (6.8)	8908 (3.5)
Family history of GI cancer		17 (2.2)	4867 (1.9)
Asthma		106 (13.8)	38,698 (15.2)
*H. pylori* infection		27 (3.5)	11,365 (4.5)
Gastro-oesophageal reflux		100 (13.0)	32,720 (12.9)
Gallstones		41 (5.3)	12,690 (5.0)
Family history of diabetes		143 (18.6)	72,791 (28.7)
Hypertension		423 (55.1)	119,653 (47.2)
Breast cancer		24 (3.1)	4505 (1.8)
Prostate cancer		17 (2.2)	3586 (1.4)
Abdominal pain^a^		83 (10.8)	8012 (3.2)
Back pain^a^		53 (6.9)	1997 (5.5)
Constipation^a^		21 (2.7)	2392 (0.9)
Diarrhoea^a^		18 (2.3)	2820 (1.1)
Heartburn^a^		18 (2.3)	2877 (1.1)
Indigestion^a^		17 (2.2)	2517 (1.0)
Jaundice^a^		14 (1.8)	122 (<0.1)
Nausea^a^		11 (1.4)	838 (0.3)
Tiredness^a^		33 (4.3)	8615 (3.4)
Weight loss^a^		29 (3.8)	1570 (0.6)
Proton pump inhibitor use		260 (33.9)	65,881 (26.0)
Bisphosphonate use		43 (5.6)	5845 (2.3)
Aspirin use		174 (22.7)	34,213 (13.5)
Statin use		360 (46.9)	88,485 (34.9)
Calcium channel blocker use		204 (26.6)	56,602 (22.3)
Digoxin use		13 (1.7)	4723 (1.9)
HbA1c (mmol/mol)	Mean (SD) Not recorded (%)	66.9 (24.1) 117 (15.5)	61.7 (20.4) 34,241 (13.5)
Haemoglobin (g/L)	Mean (SD) Not recorded (%)	140.1 (14.2) 55 (7.2)	142.9 (15.2) 30,284 (11.9)
Bilirubin (μmol/L)	Mean (SD) Not recorded (%)	10.8 (6.0) 45 (5.9)	10.1 (5.2) 21,486 (8.5)
ALT (units/L)	Mean (SD) Not recorded (%)	27.4 (15.3) 89 (11.6)	34.5 (19.3) 34,044 (13.4)
Creatinine (μmol/L)	Mean (SD) Not recorded (%)	81.2 (22.6) 17 (2.21)	79.8 (20.7) 9926 (3.9)
C-reactive protein (mg/dL)	Mean (SD) Not recorded (%)	11.8 (19.6) 451 (58.8)	10.8 (19.8) 171,022 (67.4)
Erythrocyte sedimentation rate (mm/h)	Mean (SD) Not recorded (%)	19.2 (19.8) 474 (62.6)	16.8 (16.8) 176,814 (69.7)
Platelets (billion platelets/L)	Mean (SD) Not recorded (%)	240.5 (73.3) 55 (7.2)	255.3 (70.0) 30,675 (12.1)
White blood cell count (10^9^/L)	Mean (SD) Not recorded (%)	7.8 (2.4) 56 (7.4)	7.8 (2.2) 30,283 (11.9)

Candidate predictor variables that had fewer than 10 recorded events were deselected from the modelling due to considerations of model stability and precision. This included cholangitis, pancreatic cyst, coeliac disease, Cushing’s syndrome, Hepatitis C, HIV/AIDs, fever, flatulence, abdominal mass, bowel change GI bleeding, dark urine, vomiting, tiredness, H2 blocker, steatorrhea, itching, dysphagia, appetite loss, abdominal distension. Medication exposure is defined as at least 3 prescriptions within 12 months prior to the prediction date. BMI and blood test values are latest recorded within the 3 years preceding the prediction date. Numbers are *n* (%) unless otherwise indicated.

^a^Symptom recorded within 6 months prior to the prediction date.

### Missing data

There were missing data for smoking status, self-reported ethnicity, alcohol intake, Townsend deprivation score, BMI, and the selected blood markers. Under the missing-at-random assumption, multiple imputation with chained equations was used to generate 10 imputed datasets. The extent of missing data for candidate predictors is summarised in Table 1, where relevant. The imputation model included the outcome, all candidate predictors, and the Nelson–Aalen cumulative hazard estimate [16]. BMI was imputed on the log scale for normality and back-transformed for modelling. Multiply imputed data were used for model fitting and all subsequent analyses.

### Descriptive analyses

Percentages of individuals that underwent an abdominal computed tomography (CT) scan or abdominal ultrasound (USS) within one month of T2DM diagnosis (as recorded in the HES database) were calculated to assess compliance with NICE guidance. Crude incidence rates of the outcome of interest were estimated (overall and by geographical region).

### Model development and performance assessment

The analysis strategy was to fit models to the entire study cohort, and then assess their performance using internal–external cross-validation (IECV) [17] accounting for clustering by practice. For IECV, the dataset was non-randomly split by geographical region in England (*n* = 10), then the model was iteratively fit to data from all-but-one region and evaluated on the held-out region. This was repeated for each region, so that predictions were generated for all individuals whilst ‘held out’. IECV can provide a stronger assessment of the performance and transportability of a model to new samples than a single random split. Random splitting yields two non-independent sub-datasets with similar distributions of predictors and outcomes, reduces the sample size for fitting a model and provides only a small portion of data for evaluation [15]. In contrast, IECV enables use of all the available data to fit a model and evaluate it, and emulates the process of fitting a model and applying it to a new, structurally different population [15].

Using the complete case data, the best functional forms for continuous variables (i.e. age, HbA1c, platelet count, ALT, bilirubin, haemoglobin and serum creatinine) were explored using fractional polynomials (FPs) with up to two powers [18]. FPs are a flexible approach to modelling non-linearities in continuous variables, and ‘two powers’ refers to up to two coefficients being used to model the variable–outcome relationship, potentially raised to powers that are integers or fractions. These FP terms were used in the Cox modelling. A Cox model was fit using all candidate predictors, including pre-specified interactions between age and sex. Continuous variables and interactions associated with *p* < 0.01, and binary variables associated with an exponentiated coefficient (hazard ratio) >1.1 or <0.9 with *p* < 0.01 were selected for inclusion in the final model where they were clinically plausible. This predictor selection approach considers both the statistical and clinical significance of predictor-outcome associations. The final Cox model was then fit using these selected predictors. Rubin’s rules were used to combine coefficients and the baseline survival function at 2 years across the imputed datasets [19].

To permit benchmarking, the same variables selected for the Cox model were used for the machine learning modelling. Jack-knife pseudo-observations [20, 21] for the Kaplan–Meier failure probability at 2 years were estimated in the full cohort data. For each individual, these pseudo-observations can be interpreted as their ‘contribution’ to the Kaplan–Meier failure function at the time of interest, and can be used as the outcome variable in models that output probabilistic predictions using time-to-event, censored data. In the machine learning analyses, these were used as a continuous outcome variable for both the XGBoost and neural network models [21]. As Rubin’s rules cannot be applied to machine learning models, the XGBoost and neural network models were fit to the stacked imputed datasets. Continuous variables were left on their regular scale (XGBoost), or min–max scaled (neural network). For both, categorical variables were converted to dummy variables. The XGBoost model had a ‘reg:squarederror’ objective and a root mean squared error evaluation metric. The neural network was feedforward network with fully connected layers, ReLU activation functions in each hidden layer, used the Adam optimiser, and had a single output node with a linear activation function. The root mean squared error between observed and predicted pseudo-observations was used as the loss function [21]. Hyperparameter tuning used Bayesian Optimisation (50 iterations) and fivefold cross-validation—the optimal configurations (Supplementary Table 1) identified were used to fit the XGBoost and neural network models to the entire data.

Using the individual-level predictions generated during IECV, region-level estimates of the Harrell’s *C*-index, calibration slope, and calibration-in-the-large for each model [22] were pooled using random effects meta-analysis with the Hartung–Knapp–Sidik–Jonkmann method [23]. This also provided a 95% prediction interval (PI), which provides an estimate of the range of performance that may be expected if the models were applied to a similar population. For the Cox model, Royston & Sauerbrei’s *D* statistic and *R*^2^ were estimated [24]—these are not estimable for the machine learning approaches. In each iteration of IECV, hyperparameter tuning was repeated for the machine learning models to provide ‘nested’ cross-validation.

Pooled predictions generated from IECV were used to generate calibration plots (based on ‘risk groups’ and smoothed) and perform decision curve analysis, which compared the clinical utility of all models. The sensitivity and specificity of each model was assessed based on using cut-offs at the highest 1%, 5%, and 10% of their predicted risk distributions.

### Minimum sample size

Before the study, we determined that with a target prediction horizon of 2 years, assuming a conservative 0.3% diagnosis rate of pancreatic cancer, 100 candidate predictor parameters, a Cox–Snell *R*^2^ of 0.0105 (15% of maximum permitted, 0.07), and a mean follow-up of 2 years, a minimum of 85,214 individuals with type 2 diabetes were required (5.11 events per predictor parameter) to fit the Cox proportional hazards prediction model [25]. No clear guidance exists regarding minimum sample size for machine learning models.

### Statistical software

Data management, statistical analyses and model evaluation steps were performed using Stata V17. Machine learning model building and IECV used R (packages: keras, xgboost [both with GPU support], and ParBayesianOptimization).

### Patient and public involvement

Pancreatic Cancer UK Research Involvement Network (RIN) lay members who have a lived experience of pancreatic cancer or have cared for somebody affected helped to develop research questions and assisted in the writing of lay summary for this study.

## Results

### Baseline characteristics and incidence rates

The final study cohort comprised 253,766 individuals with a new diagnosis of T2DM—baseline characteristics are summarised in Table 1. Restricting follow-up to a maximum of 2 years after the prediction date (diagnosis plus 2 weeks), there were 767 incident pancreatic cancer diagnoses within 442,347.8 person-years, with a crude incidence rate of 17.34 per 10,000 person-years (95% CI: 16.16–18.61). Crude incidence rates for females and males were 17.88 (95% CI: 16.08–19.89) and 16.93 (95% CI: 15.40–18.62) per 10,000 person-years, respectively. The yield of using multiple linked databases for outcome ascertainment is shown in Supplementary Table 2. Ethnic group- and region-specific crude incidence rates are summarised in Supplementary Tables 3 and 4, respectively.

Of the 253,766 individuals included, 1570 (0.62%) had weight loss recorded in their primary care record within the preceding 6 months (SNOMED codes); 763 (0.30% of cohort) were aged 60+ years and had weight loss recorded and would therefore meet NICE criteria for referral for urgent imaging. Of the study cohort, 415 (0.16%) underwent an USS (standard external, or endoscopic ultrasound of the pancreas) within the 30 days of the prediction date, and 998 (0.39%) underwent CT imaging within the same timeframe. In the sub-group meeting NICE criteria (*n* = 763), 10 (1.31%) underwent an ultrasound, and 17 (2.23%) underwent CT within 30 days of the prediction date. Within 1 year of the prediction date, 1924 individuals (0.76%) had an USS (median time to scan 126 days, IQR: 52–236 days), and 7013 (2.76%) had an abdominal CT (median time to scan 158 days, IQR: 74–264 days).

### Model development

Non-linear (FP) terms were selected for age and HbA1c in the Cox proportional hazards model (Supplementary Fig. 2). There were no significant interactions between age and predictor variables included in the final model. The final model included age, sex, BMI, prior venous thromboembolism, digoxin prescription, HbA1c, ALT, creatinine, haemoglobin, platelet count, and the presence of the following symptoms within 6 months prior to the prediction date: abdominal pain, weight loss, jaundice, heartburn, indigestion and nausea.

**Fig. 1:**
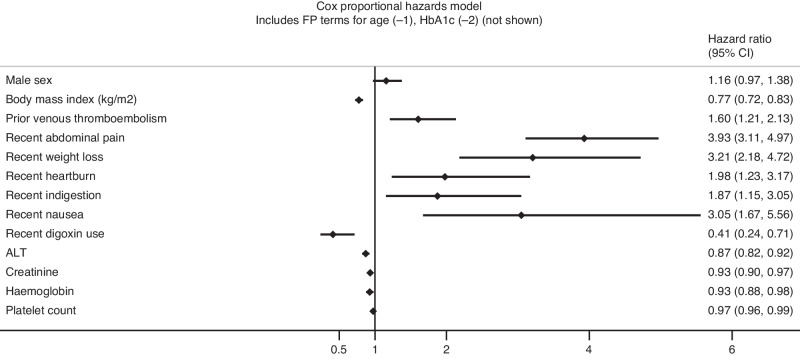
Forest plot demonstrating the final Cox proportional hazards model as its exponentiated coefficients (hazard ratios, with 95% confidence intervals). The full model including the baseline survival term is detailed in the supplement. The term for jaundice is not plotted due to the magnitude of the hazard ratio affecting visualisation on similar scale as the other predictors. The hazard ratios for body mass index correspond to a per-5 unit increase, whereas the hazard ratios for ALT, creatinine, haemoglobin and platelet count correspond to a per-10 unit increase.

The final Cox model is displayed as its exponentiated coefficients (hazard ratios, with 95% CIs) in Fig. 1. The full model (as coefficients, including baseline survival term) is summarised in Supplementary Table 5.

### Model evaluation

**Fig. 2:**
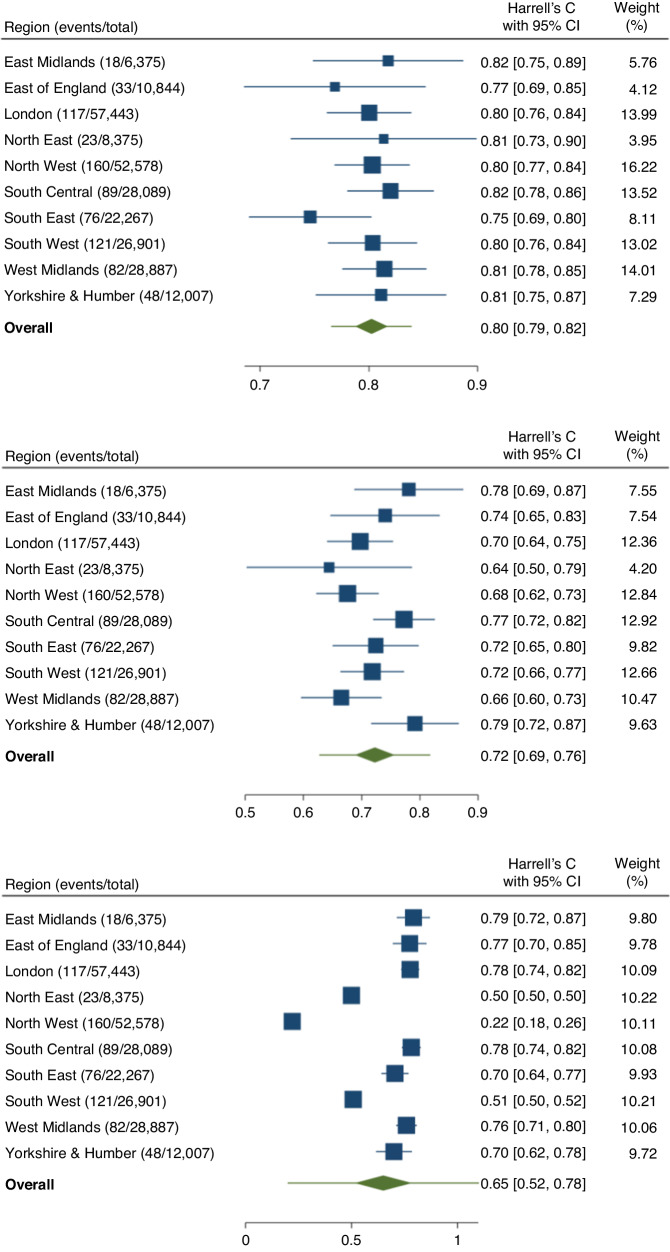
Regional-level estimates and pooled overall meta-estimates for Harrell’s *C*-index for each model. The green diamond refers to the 95% confidence interval for the pooled meta-estimate, the horizontal lines through these correspond to the 95% prediction interval.

Summary performance metrics estimated after IECV for all models are shown in Table 2. Region-level and meta-analysis pooled estimates of Harrell’s *C*-index and calibration slope for all models are summarised in Fig. 2 and Supplementary Fig. 3, respectively.

**Table 2:** Performance metrics with corresponding 95% confidence intervals for each model.

Metric	Estimate (95% confidence interval) [95% prediction interval]		
	Cox proportional hazards model	XGBoost	Neural Network
Harrell’s *C* index	0.802 (0.787 to 0.817) [0.766 to 0.839]	0.723 (0.689 to 0.756) [0.628 to 0.817]	0.650 (0.516 to 0.784) [0.202 to 1.000]
Calibration slope	0.980 (0.897 to 1.062) [0.778 to 1.182]	1.180 (1.056 to 1.305) [0.781 to 1.580]	1.855 (−0.945 to 4.654) [−7.552 to 11.261]
Calibration-in-the-large	−0.020 (−0.103 to 0.062) [−0.222 to 0.182]	0.180 (0.056 to 0.305) [−0.219 to 0.580]	0.855 (−1.945 to 3.654) [−8.552 to 10.261]
Royston & Sauerbrei’s *D*	1.880 (1.768 to 1.993) [1.629 to 2.131]	–	–
Royston & Sauerbrei’s *R*^2^	46.0% (43.1% to 48.9%) [39.3% to 52.7%]	–	–

For the Cox and XGBoost models, these were estimated using random-effects meta-analysis following internal–external cross-validation, which also provided a 95% prediction interval.

**Fig. 3:**
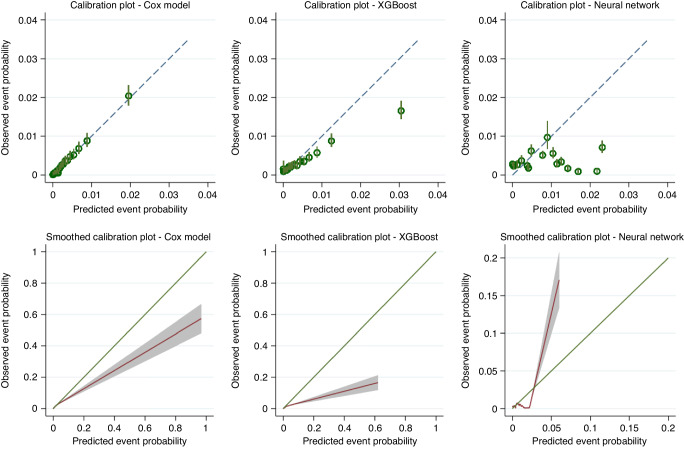
Calibration plots demonstrating the alignment between predicted and observed risks for each model using two approaches: top row = grouped by 20th of predicted risk; bottom row = smoothed plots generated by plotting a running smoother through predicted values and observed pseudo-observations for the Kaplan-Meier failure probability at 2 years. Predicted probabilities are those generated during internal–external cross-validation.

The Cox model showed the highest discrimination with a Harrell’s *C*-index of 0.802 (95% CI: 0.787–0.817, 95% PI: 0.766–0.839) compared to XGBoost (0.723, 95% CI: 0.689–0.756, 95% PI: 0.628–0.817) and the neural network model (0.650, 95% CI: 0.516–0.784). The Cox model was well calibrated on summary metrics: calibration slope 0.980 (95% CI: 0.897–1.062, 95% PI: 0.778–1.182), and appeared well calibrated on the risk group calibration plot (Fig. 3). The smoothed calibration plot showed some over-estimation of those at the very highest predicted risks.

The XGBoost model appeared systematically miscalibrated on summary measures (e.g. slope 1.180, 95% CI: 1.056–1.305, 95% PI: 0.781–1.580), which was also visible on the calibration plots (Fig. 3). The neural network had unstable performance during the IECV process—this manifested as low point estimates for Harrell’s *C* and summary calibration metrics for individual regions, with wide confidence and prediction intervals for the pooled meta-estimates.

The Cox model had a Royston & Sauerbrei’s *D*-statistic of 1.880 (95% CI: 1.768–1.993, 95% PI: 1.629–2.131), and it explained 46.0% of variation in time to pancreatic cancer diagnosis (95% CI: 43.1–48.9%, 95% PI: 39.3–52.7%)—see Supplementary Fig. 4.

### Model sensitivity and clinical utility

The top 1%, 5% and 10% of the predicted risks from IECV for the Cox model comprised 12.51%, 31.02%, and 44.06% of all pancreatic cancers diagnosed within 2 years, respectively. This was higher than for the XGBoost model (Supplementary Table 6): the top 10% of predicted risks from IECV for the XGBoost model captured 38.72% of all events. The current decision rule suggested by NICE (refer if aged 60+ years with recent weight loss) had a sensitivity of 3.53% overall, and a sensitivity of 3.95% in the over 60s (Supplementary Table 7). Specificity was similar at each threshold examined for the three models (Supplementary Table 6).

**Fig. 4:**
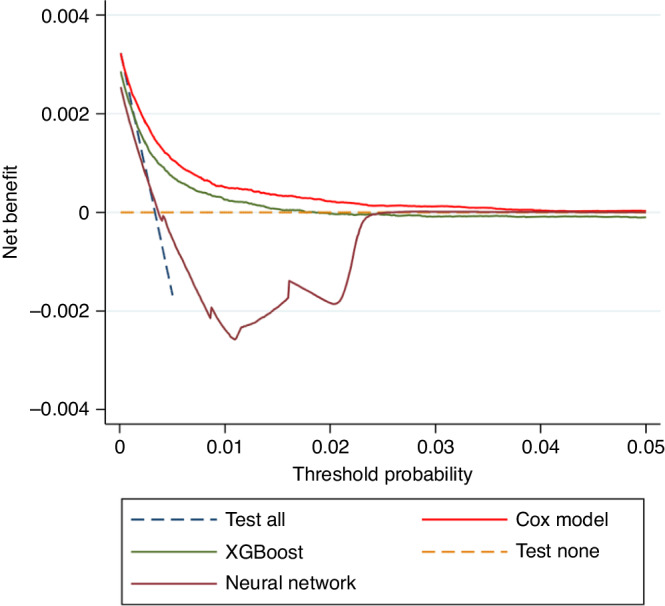
Decision curve analysis comparing the clinical utility (net benefit) of each model developed, compared to ‘test all’ and ‘test none’ strategies. These are plotted using the predictions obtained through internal–external cross-validation.

Decision curve analysis compared model clinical utility up to a threshold probability of 0.05 (5%, Fig. 4). The Cox model was associated with the highest net benefit—this was also better than the ‘test all’ strategy, which is clinically unfeasible due to logistical issues inherent to obtaining fast-track abdominal imaging in all individuals newly diagnosed with T2DM. This suggests that the Cox model was the most clinically useful model and could be beneficial in decision making regarding referrals for further investigation.

## Discussion

This study developed three clinical prediction models to estimate the 2-year risk of pancreatic cancer diagnosis in people aged 30-85 years with newly diagnosed T2DM (which as aforementioned, may in some cases be type 3c). The Cox model offered the highest discrimination, was well calibrated on summary metrics, explained 46% of variation in time to pancreatic cancer diagnosis, and was associated with the highest net benefit. The top 1% and 10% of predicted risks from the Cox model captured 12.51% and 44.06% of all subsequent pancreatic cancer cases within 2 years, respectively, compared to a 3.95% sensitivity with age and weight-loss-based decision rules currently recommended by NICE [6].

The ability to establish a pancreatic cancer diagnosis up to 2 years or even months earlier could have significant impacts. If disease was detected at an earlier, resectable stage, FOLFORINOX plus surgical resection has been shown to offer a 5-year survival of approximately 40% [26]. Furthermore, patients that are detected earlier with a more favourable performance status may be more able to tolerate cytotoxic chemotherapy, more able to tolerate multiple lines of therapy, and those with a lower burden of stage 4 disease could be eligible for multimodal therapy [27]. This is important as up to half of pancreatic cancer cases are not suitable candidates for chemotherapy due to performance status.

A strength of the study is the use of the population-representative QResearch database and its linkages to national cancer registry, secondary care, and mortality register data; this enabled the derivation of a large cohort and improved ascertainment of predictor values and outcomes. This use of routinely collected general practice data avoided recall, selection and respondent biases. Another strength was the IECV framework, which enabled use of all available data for model development, evaluation, and comparisons between modelling methods, as well as estimating model transportability.

Limitations of the study include the nature of data in routinely collected primary care databases such as QResearch, and the way in which this is obtained. The completeness and ascertainment of measurements, diagnoses and prescriptions data points are reliant on clinical coding by individual healthcare practitioners. As we did not have access to the full ‘free text’ clinical notes, the reliance on practitioners entering SNOMED codes to define such variables needs to be considered. For example, weight loss may be mentioned by an individual during a consultation, but this may not be coded in the notes. There may be variation in the proclivity of individual practitioners to input clinical codes. Further, there may be misclassification bias in the use of such data, e.g. medication being prescribed by a clinician which was not taken by the recipient, as well as information bias. The study is also limited by the lack of formal adjudication of outcomes. Other limitations include the potential bias from missing data, although multiple imputation was used to minimise this. Missing data also presents considerations for potential later model deployment—while multiple imputation offers a principled approach for model development and validation, this may not be plausible for clinical deployment. If the final model was to deployed clinically, considerations include whether missing data is not permitted (i.e. the clinician would need to ask the patient/obtain measurements for the missing values), or if missingness would be permitted (i.e. the model would be run ‘in the background’, in which case, regression imputation could be considered, or use of age- and sex-standardised reference values used). Markers of genetic predisposition such as polygenic risk scores [28] were not possible to include given the nature of the routinely collected primary care datasets, however, the clinical prediction model developed here is intended to only use such data. The added ‘yields’ of such predictors beyond clinical variables are non-uniform across other integrated modelling studies [29]. Lastly, this study was not (and could not be) a formal comparison of ‘statistical versus machine learning models’; rather, it sought to identify the best performing model from a set of techniques that either implicitly or have been adapted to handle censored, time-to-event data, within a low-dimensional setting. There is optimism in the literature that machine learning approaches could offer benefits to clinical prognostication beyond ‘classical’ regression methods [30], but others have cautioned against ‘hype’ [31], limitations of some techniques in handling time-to-event data or those with rare outcomes, ‘spin’ in the reporting of some new algorithms, and crucially, fairness of comparisons between modelling approaches [32]. Not every clinical problem requires a complex algorithm, and indeed, the Cox model selected for the final risk prediction equation performed well using a relatively small set of clinical predictors and can be reported transparently.

This study is the largest yet to develop and validate a risk prediction model for estimating the risk of pancreatic cancer in people with new-onset T2DM, but not the first to do so.

The ‘END-PAC’ 3-variable points-based score was developed and internally validated using a small sample size, with only 64 pancreatic cancer cases in the development set, and only 9 events in the validation set [8]. An external validation study used larger datasets (up to 99 cancer cases) [15], reporting an AUC of 0.75, and a sensitivity of 62.6%, specificity of 78.5%, and positive predictive value of 2.0% in those with 3 points or higher. Assessment of this points-based score has been focused on sensitivity, specificity and predictive values on binning patients into points-based ‘risk groups’, rather than our approach which considered discrimination, calibration across the spectrum of predicted risks, and clinical utility.

Prior to the current study, the largest other modelling paper was that of Boursi, et al. [33] which included 109,385 patients with new onset diabetes and 390 incident cases of pancreatic cancer. Whilst this model was also developed using UK-based general practice data, it has some methodological limitations including its incorrect use of logistic regression for a ‘binary outcome’ (the model has a 3-year prediction horizon) [10]. The Boursi study used data from 1995 to 2013, whereas the present study used a more recent cohort from linked data sources offering more robust outcome ascertainment, assessed a broader range of candidate predictors, and considered non-linearities. The point estimate for the discrimination metric was slightly higher in the Boursi study (AUC 0.82, 95% CI: 0.75–0.89)—this should be considered in the context of the Boursi study not accounting for censoring, that the CIs for Harrell’s *C* in the present study overlap with the results of Boursi et al. and that our study additionally considered calibration and the usefulness of the models on clinical decision making through decision curve analysis. The present study using the QResearch database and linked data assets was not able to not perform an external validation of the developed models—a future, comparative external validation of the Boursi et al. model with the Cox model from the present study in an independent dataset, potentially by an independent group, could be of interest.

Our analyses of model sensitivity and specificity were performed at a relatively small number of thresholds. Naturally, the trade-offs between true positives, false negatives and false positives will vary as the model’s probabilistic predictions are dichotomised to form different risk groups. These thresholds were used to illustrate model performance in the context of a broad range of metrics such as clinical utility and are not set or recommended cut-offs for any future model use. The full ramifications on clinical and cost-effectiveness of using different thresholds should be explored in future health economic modelling studies. Our study sought to explore a set of algorithmic approaches to develop a clinically useful model for a group at higher risk of pancreatic cancers—follow-on work from cost-effectiveness analyses will be useful to identify the optimal ways in which could be used.

By comparatively developing and validating models using three methods, this study identified that the best model was obtained using Cox proportional hazards modelling. This final clinical prediction model could have utility in informing pathways for expedited diagnosis of pancreatic cancers in adults with new-onset T2DM and could do so more effectively than current ‘rules-based’ referral guidelines. Further evaluation such as external validation and health economic assessment is warranted prior to implementation, such as modelling the effects of different risk thresholds for triggering imaging referrals and the costs associated therewith.

### Supplementary information

Supplementary file

## Data Availability

Statistical code and other analysis code can be made available upon request to the corresponding author.

## Data availability

Data collected for this study cannot be made routinely available to others due to the regulations for access to QResearch data. Regulations regarding access to the QResearch database are detailed at https://www.qresearch.org/information/information-for-researchers/ and include approval of a proposal by the QResearch Scientific Committee. The protocol has been published previously (reference #12).
