# Supplementary material for: Predicting the risk of pancreatic cancer in adults with new-onset diabetes: development and internal–external validation of a clinical risk prediction model
Source: Br J Cancer. 2024 May 3;130(12):1969–78. doi: 10.1038/s41416-024-02693-9 (PMC11183048; doi:10.1038/s41416-024-02693-9)
Supplement: Supplementary file 1 — Supplementary file [file 41416_2024_2693_MOESM1_ESM.docx]

n=367,542 (Initial cohort of people with a recorded diagnosis of type 2 diabetes, 2010-2021)

Exclude: T2DM diagnosed prior to cohort entry date (n=91,154)

n=276,388

Exclude: exited cohort prior to prediction date (n=1,497)

n=274,891

Exclude: recorded prior diagnosis of pancreatic cancer (n=631)

n= 274,260

Exclude: diabetes medications before date of T2DM diagnosis (n= 20,494)

n=253,766 (Final cohort)

**Supplementary Figure 1:** Participant flow chart


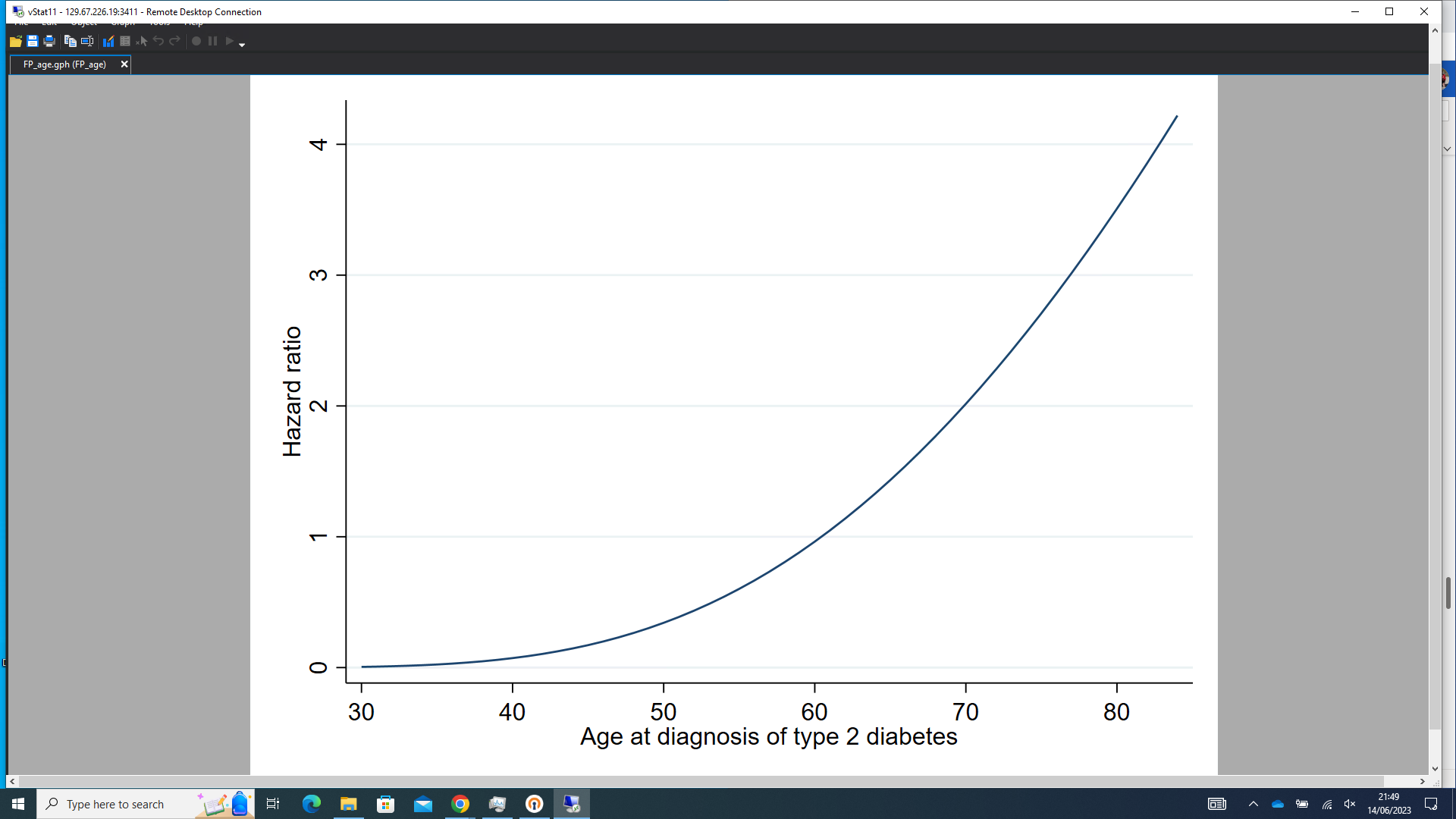


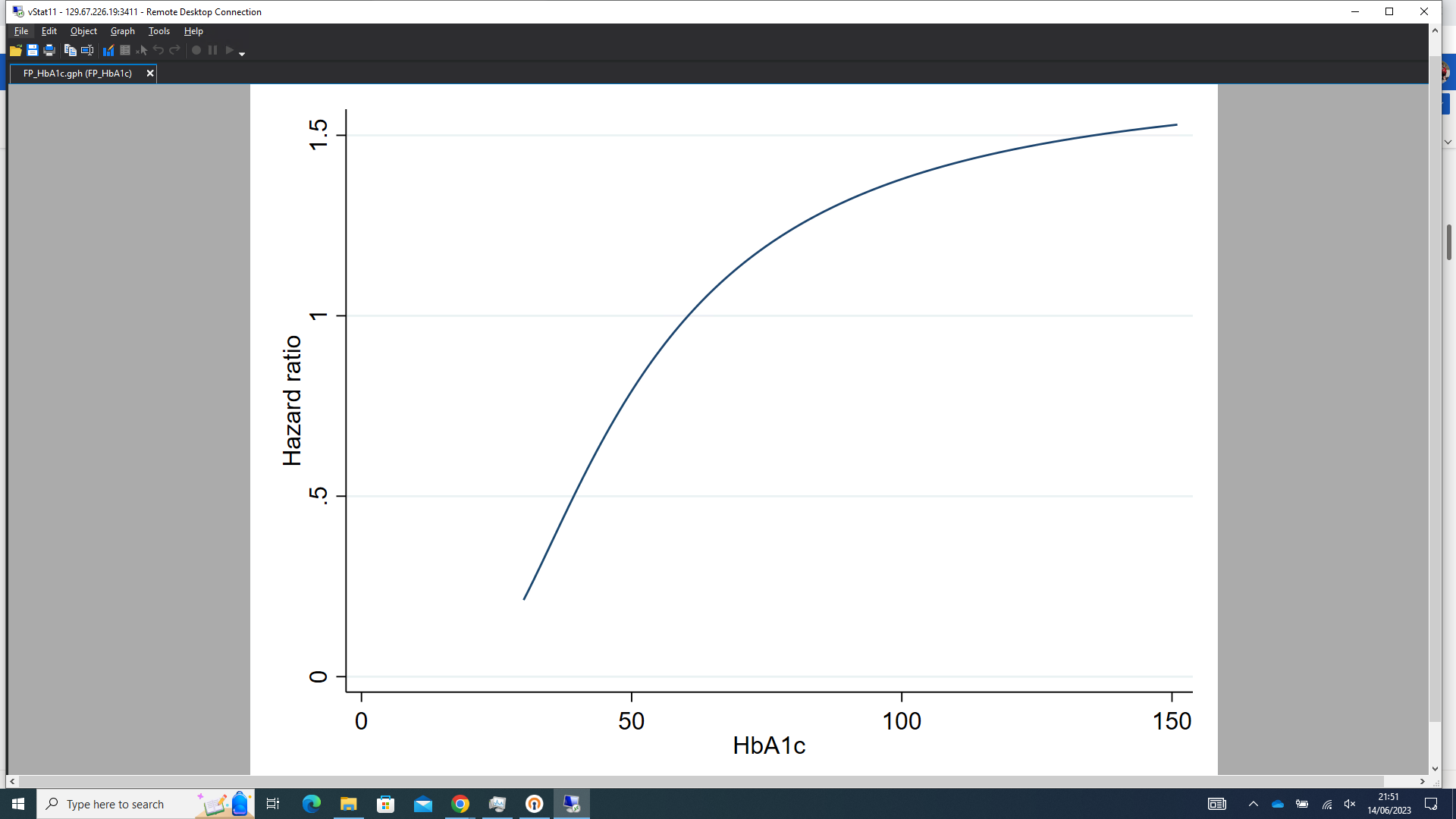


**Supplementary Figure 2:**  Fractional polynomial forms identified for age (-1) and HbA1c (-2) identified for the Cox proportional hazards model.


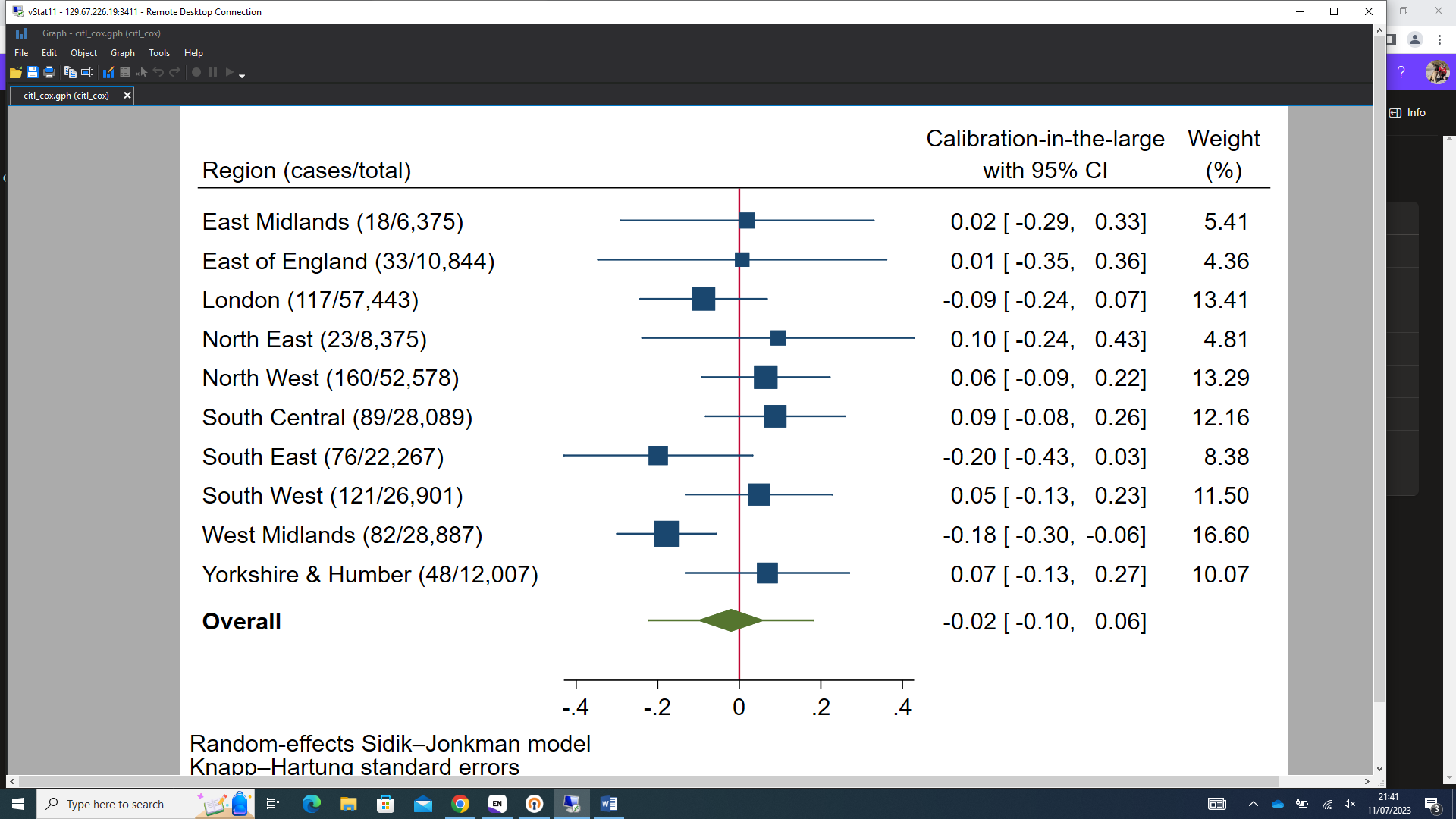


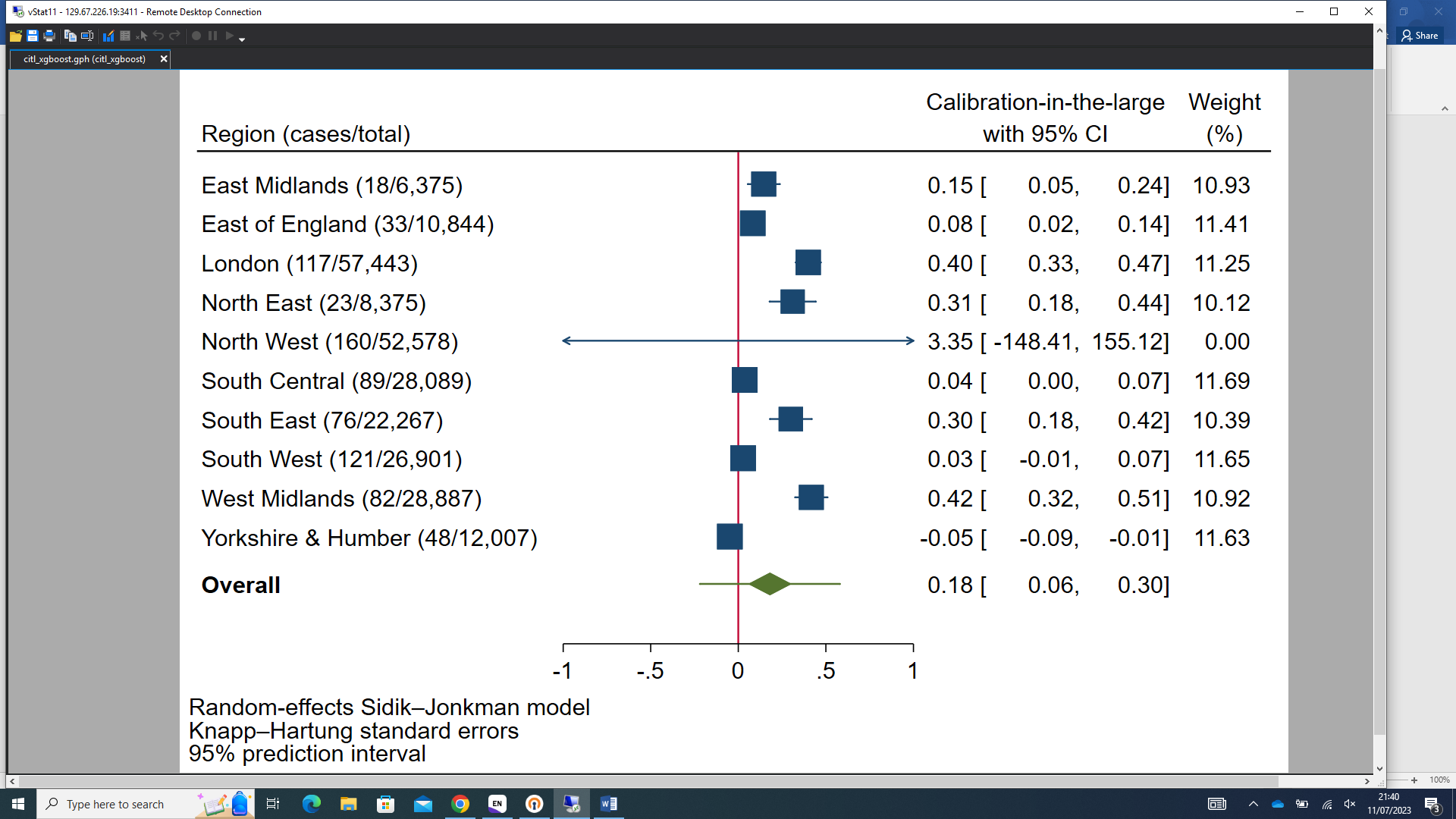


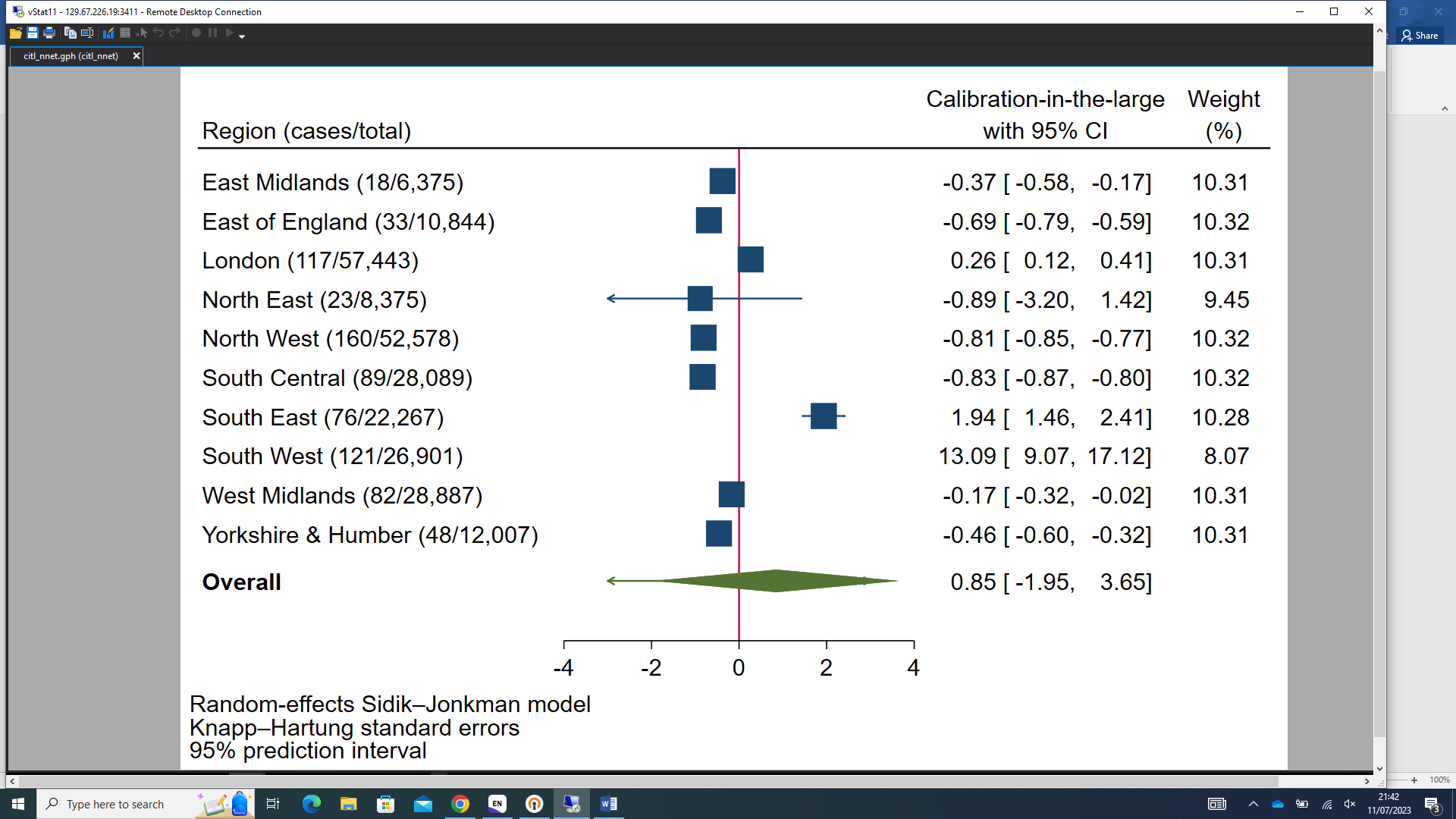


**Supplementary Figure 3:** Region-level estimates for calibration-in-the-large for the Cox proportional hazards (top), XGBoost (middle) and neural networks (bottom) models. Overall meta-estimate with 95% confidence and 95% prediction intervals estimated following internal-external cross validation.


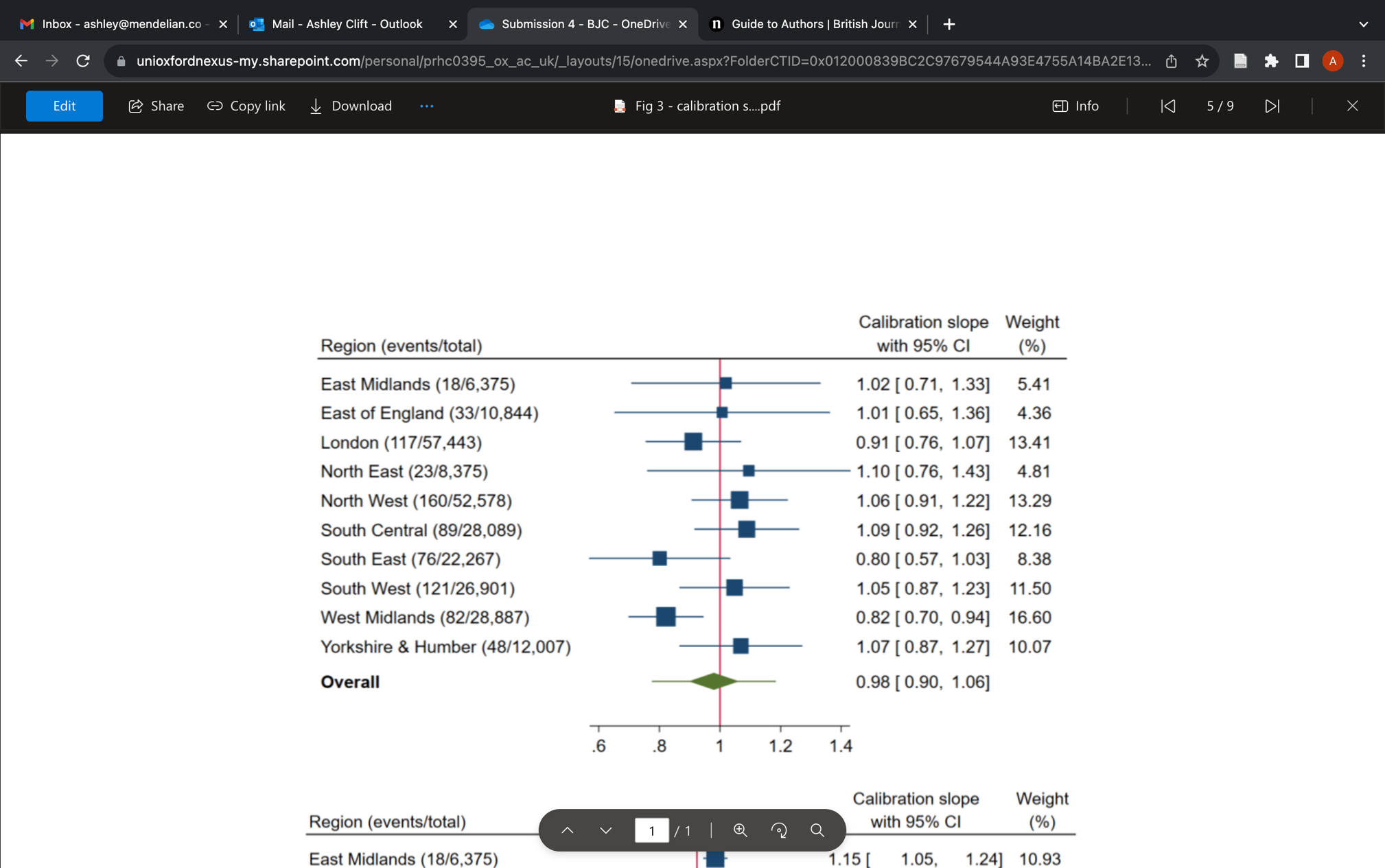


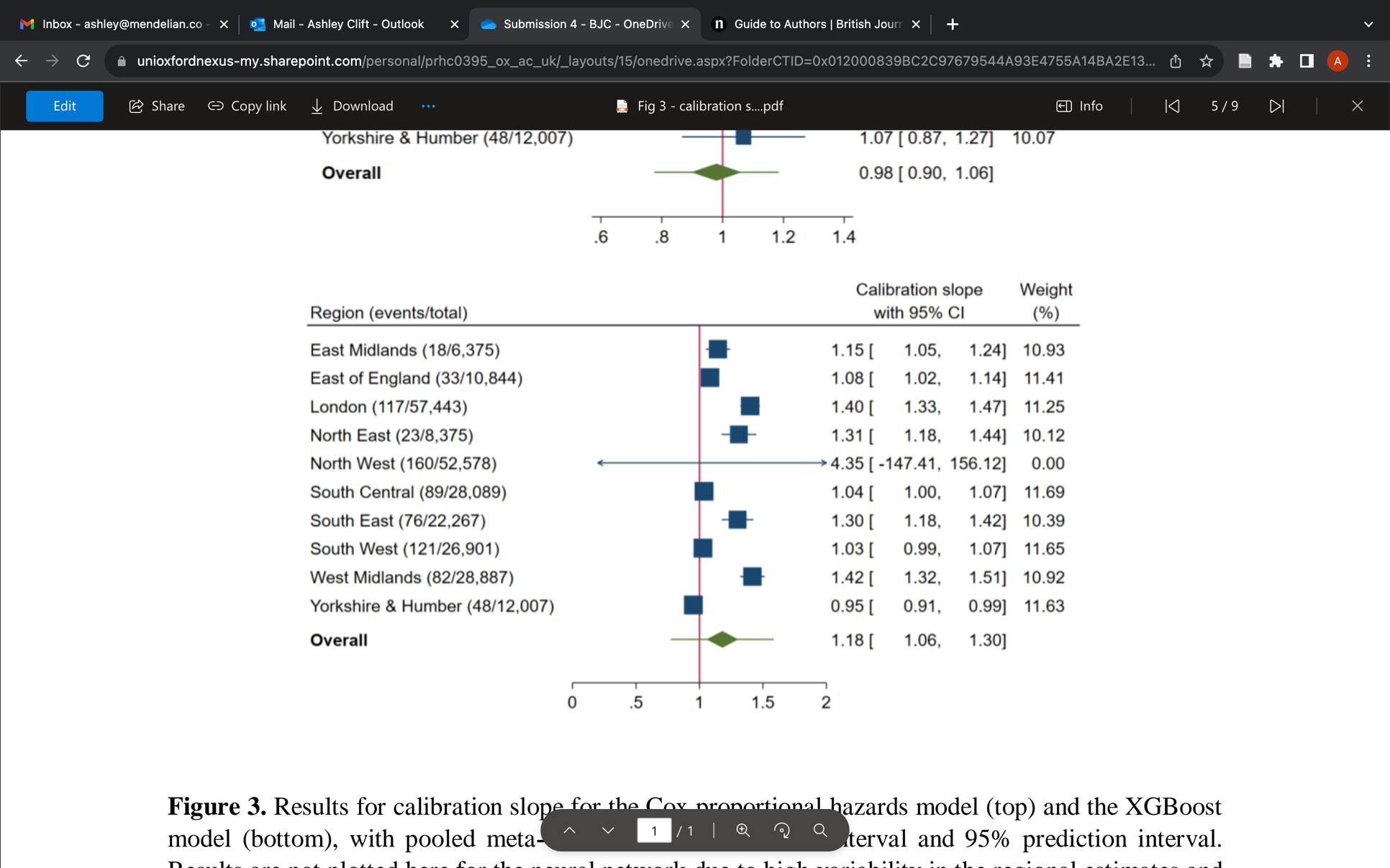


**Supplementary Figure 3.** Results for calibration slope for the Cox proportional hazards model (top) and the XGBoost model (bottom), with pooled meta-estimate, 95% confidence interval and 95% prediction interval. Results are not plotted here for the neural network due to high variability in the regional estimates and confidence intervals.


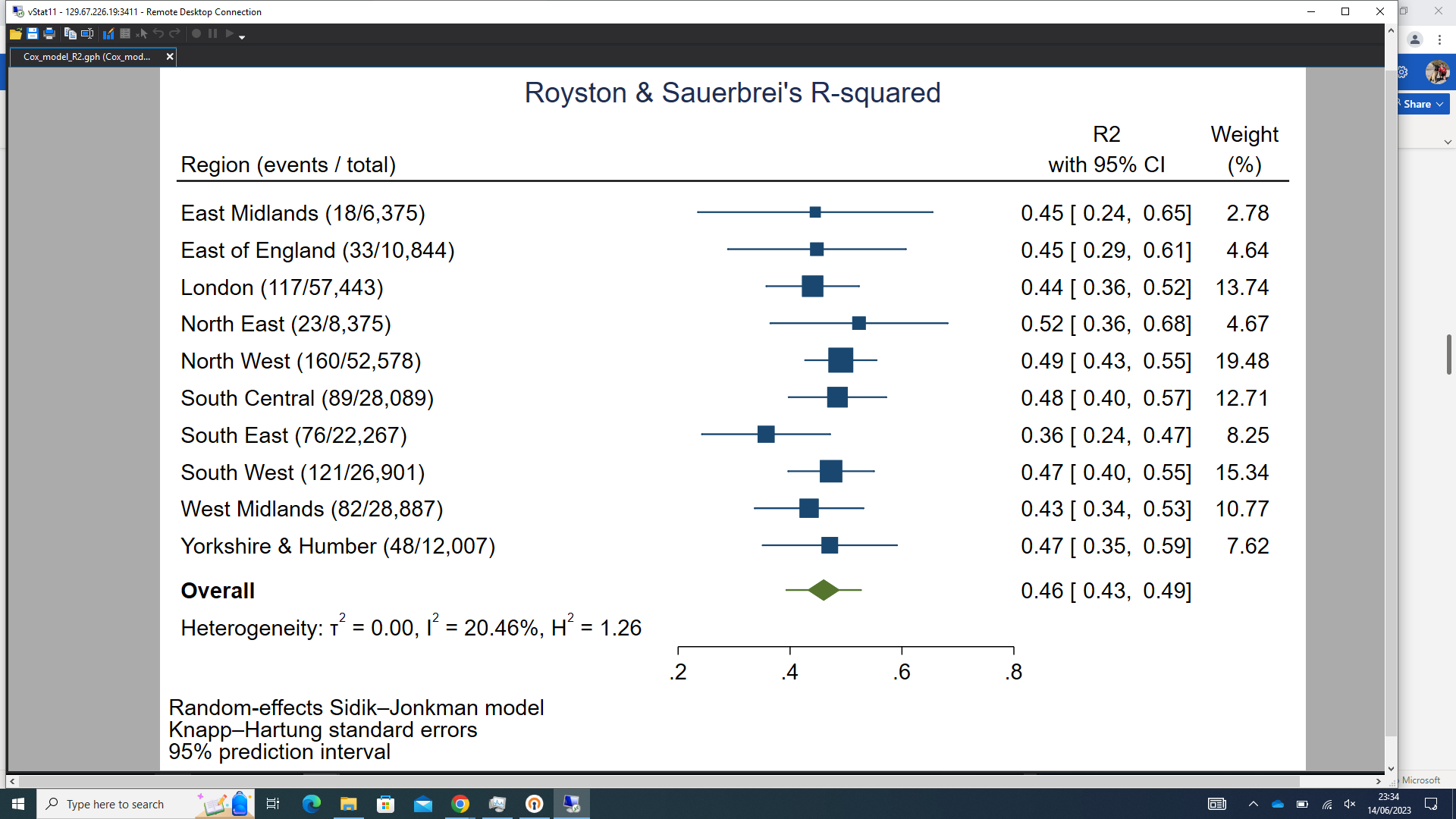


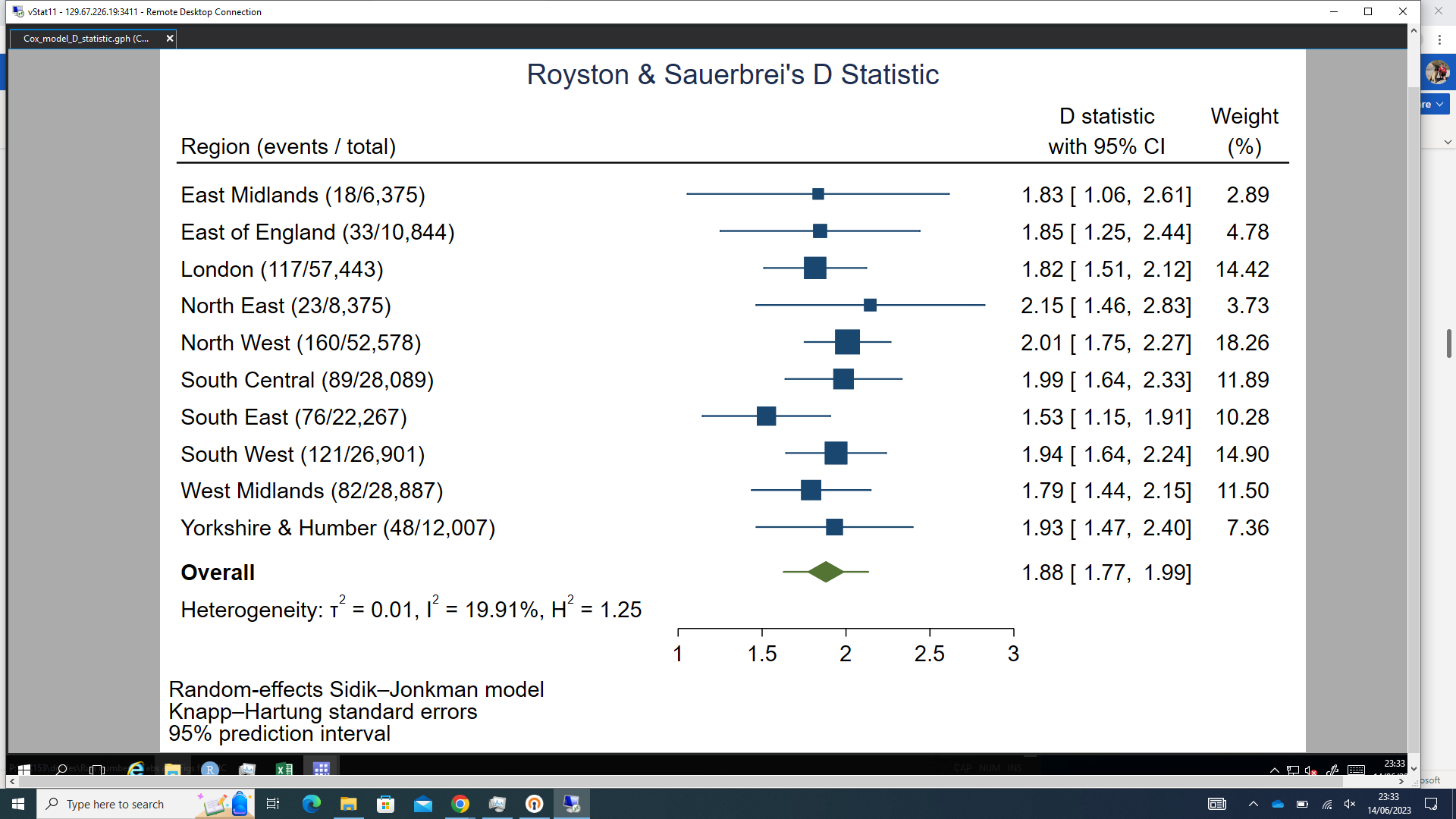


**Supplementary Figure 5:** Region-level estimates for Royston & Sauerbrei's R^2^D (top) and D-statistic (bottom) for the Cox proportional hazards model. Overall meta-estimate with 95% confidence and 95% prediction intervals estimated using internal-external cross validation.

| Model | Basic architecture | Hyperparameters tuned | Range explored | Selected value |
| --- | --- | --- | --- | --- |
| XGBoost | Tree-based booster with GPU_hist  Mean squared error objective  RMSE evaluation metric | Maximum tree depth  Learning rate (eta)  Number of boosting rounds  Alpha (regularisation)  Gamma (regularisation)  Lambda (regularisation)  Column sampling by tree  Column sampling by level | 1 to 6  0.0001 to 0.1  0 to 1000  0 to 20  0 to 20  0 to 20  0.1 to 0.8  0.1 to 0.8 | 2  0.158  421  0  0  20  0.800  0.800 |
| Neural network | Feed-forward ANN  Fully connected layers (batch normalisation each layer)  ReLU activation functions in hidden layers  Adam optimiser  Single output node with linear activation  RMSE loss function  Batch size 256 | Number of hidden layers  Number of nodes in each hidden layer  Number of epochs  Initial learning rate | 1 to 5  10 to 50  1 to 20  0.001 to 0.1 | 1  50  6  0.008 |

**Supplementary Table 1:** Optimal configurations for hyperparameter tuning identified with Bayesian Optimisation, and hyperparameter search spaces explored, for the two machine learning models.

| Database(s) | Number of pancreatic cancer cases within 2 years from date of type 2 diabetes diagnosis  (% of total) | Person-years of follow-up | Crude incidence rate per 10,000 person-years (95% confidence interval) |
| --- | --- | --- | --- |
| QResearch (GP data only) | 577 (75.23%) | 442,347.80 | 13.04 (12.02 to 14.15) |
| QResearch + Cancer Registry | 702 (91.52%) | 442,347.80 | 15.87 (14.74 to 17.09) |
| QResearch + Cancer Registry + HES | 748 (97.52%) | 442,347.80 | 16.91 (15.74 to 18.17) |
| QResearch + Cancer Registry + HES + ONS death register | 767 (100%) | 442,347.80 | 17.34 (16.16 to 18.61) |

**Supplementary Table 2.** Cumulative yield of using multiple linked databases for ascertainment of the outcome of interest.

| Ethnic group* | Number of incident pancreatic cancer cases | Person-years of follow-up | Crude incidence rate of pancreatic cancer diagnosis per 10,000 person-years  (95% CI) |
| --- | --- | --- | --- |
| White | 582 | 29,168 | 19.95 (18.40 to 21.64) |
| South Asian* | 20 | 48,027 | 4.16 (2.69 to 6.45) |
| Black* | 17 | 22,175 | 7.67 (4.77 to 12.33) |
| Other * | 20 | 25,765 | 7.76 (5.01 to 12.03) |
| Not recorded | 128 | 54,702 | 23.40 (19.67 to 27.83) |

**Supplementary Table 3.** Ethnic group-specific crude incidence rates of pancreatic cancer diagnosis subsequent to type 2 diabetes mellitus diagnosis. Follow-up was truncated to a maximum of 2 years from the prediction date (date of type 2 diabetes diagnosis plus 2 weeks). *These composite ethnic groups were formed due to the low event counts in more granular classifications (‘South Asian’ comprises Indian, Pakistani and Bangladeshi ethnic groups; ‘Black’ comprises Black British, Black Caribbean, Black African, and ‘Other Black’ ethnic groups; ‘Other’ comprises Chinese, Other Asian, mixed race groups, and the ‘Other Ethnic Group’ Office for National Statistics categories).

| Geographical region | Number of incident pancreatic cancer cases | Person-years of follow-up | Crude incidence rate of pancreatic cancer diagnosis per 10,000 person-years (95% CI) |
| --- | --- | --- | --- |
| East Midlands | 18 | 11,264 | 15.98 (10.07 to 25.36) |
| East of England | 33 | 19,054 | 17.32 (12.32 to 24.36) |
| London | 117 | 99,745 | 11.73 (9.79 to 14.06) |
| North East | 23 | 14,711 | 15.63 (10.39 to 23.52) |
| North West | 160 | 92,361 | 17.32 (14.83 to 20.23) |
| South Central | 89 | 48,261 | 18.44 (14.98 to 22.70) |
| South East | 76 | 38,207 | 19.89 (15.89 to 24.91) |
| South West | 121 | 46,964 | 25.76 (21.56 to 30.79) |
| West Midlands | 82 | 50,590 | 16.21 (13.05 to 20.12) |
| Yorkshire & Humber | 48 | 21,192 | 22.65 (17.07 to 30.06) |

**Supplementary Table 4.** Geographical region-specific crude incidence rates of pancreatic cancer diagnosis subsequent to type 2 diabetes mellitus diagnosis. Follow-up was truncated to a maximum of 2 years from the prediction date (date of type 2 duabetes diagnosis plus two weeks).

| Parameter | Description/label | Coefficient |
| --- | --- | --- |
| Age at diagnosis of type 2 diabetes  (FP term) | X^-1^ – 0.1654610324  (X = age in years/10) | 0.07095241 |
| Sex | Female (reference)  Male | 0  0.14543252 |
| Body mass index  (kg/m^2^ ) |  | -0.05129289 |
| Venous thromboembolism | No (reference)  Yes | 0  0.47310156 |
| Abdominal pain within previous 6 months | No (reference)  Yes | 0  1.3682102 |
| Weight loss within previous 6 months | No (reference)  Yes | 0  1.162737 |
| Jaundice within previous 6 months | No (reference)  Yes | 0  3.2150278 |
| Heartburn within previous 6 months | No (reference)  Yes | 0  0.68224663 |
| Indigestion within previous 6 months | No (reference)  Yes | 0  0.62779226 |
| Nausea within previous 6 months | No (reference)  Yes | 0  1.1133372 |
| Prescribed use of digoxin | No (reference)  Yes | 0  -0.89064168 |
| HbA1c (mmol/mol)  (FP term) | X^-2^ – 2.736757656  (X = HbA1c/100) | -0.33389533 |
| ALT (units/L) |  | -0.01379719 |
| Creatinine (umol/L) |  | -0.00699232 |
| Haemoglobin (g/L) |  | -0.00776606 |
| Platelet count (10^9/L) |  | -0.00261794 |
| Baseline survival function at 2 years |  | 0.84198531 |

**Supplementary Table 5**. Coefficients and baseline survival function for the final Cox proportional hazards model.

| Predicted risk threshold (highest) |  | | | |  |  |
| --- | --- | --- | --- | --- | --- | --- |
|  | Total number of pancreatic cancer cases | Cumulative % of pancreatic cancer cases  (sensitivity) | | Specificity at predicted risk threshold | |  |
| 1% | 96 | | 12.51% 99.03% | |  |  |
| 5% | 238 | | 31.02% 95.08% | |  |  |
| 10% | 338 | | 44.06% 90.10% | |  |  |
|  | **XGBoost** | |  | |  |  |
| 1%  5%  10% | 68  195  297 | | 8.87% 99.02%  25.42% 95.01%  38.72% 90.01% | |  |  |
|  | **Neural network** | |  | |  |  |
| 1%  5%  10% | 31  83  92 | | 4.04% 99.00%  10.82%. 95.02%  11.99%. 90.00% | |  |  |

**Supplementary Table 6**. Sensitivity and specificity of the Cox, XGBoost, and neural network models. Sensitivity is estimated using the cumulative number of events (incident pancreatic cancer diagnoses within 2 years after the prediction date) captured in each of the above highest percentages of predicted risks. Specificity is estimated using the total number of true negatives (those below the risk threshold and did not have an event) divided by the total non-events in the cohort. All results are derived using the individual-level predictions from internal-external cross-validation. These three thresholds were chosen for illustrative purposes to demonstrate the performance of the model and should be interpreted in the context of other results, primarily the clinical utility analyses. These are not recommended thresholds for any future use.

| *Whole cohort* |  | Aged 60+ years, with recent recorded weight loss? | |
| --- | --- | --- | --- |
| Pancreatic cancer diagnosis within 2 years of prediction date |  | Yes | No |
|  | Yes | 26 | 741 |
|  | No | 737 | 252,262 |

| *Those aged 60+ years* |  | Aged 60+ years, with recent recorded weight loss? | |
| --- | --- | --- | --- |
| Pancreatic cancer diagnosis within 2 years of prediction date |  | Yes | No |
|  | Yes | 26 | 632 |
|  | No | 737 | 130,305 |

**Supplementary Table 7.** Two-by-two tables used to assess the **s**ensitivity of rule-based decision making as currently recommended by NICE (refer if aged 60 years and over, with recent weight loss), applied to the entire study cohort (top), and the sub-cohort aged 60 years and above (bottom). Percentage sensitivity is calculated as the number of pancreatic cancer cases correctly ‘flagged’ by the NICE criteria. In the whole cohort, the sensitivity of the NICE criteria is 3.53%, in those aged 60 years and older, it was 3.95%.

| **Age group** | **Harrell’s C index (95% CI)** | **Calibration slope (95% CI)** |
| --- | --- | --- |
| 30-39 years | 0.66 (0.179 to 1.00) | 0.596 (-0.584 to 1.777) |
| 40-49 years | 0.707 (0.565 to 0.849) | 0.681 (0.230 to 1.103) |
| 50-59 years | 0.742 (0.683 to 0.802) | 1.122 (0.906 to 1.338) |
| 60-69 years | 0.708 (0.672 to 0.744) | 0.913 (0.782 to 1.045) |
| 70+ years | 0.705 (0.677 to 0.733) | 0.958 (0.850 to 1.066) |

**Supplementary Table 8.** Summary performance metrics of the final Cox model by selected age-groups. CI = confidence interval. Due to the relatively lower number of individuals and events in the 30–39-year group, imprecision is noted.

| **Predictor** | **Royston & Sauerbrei’s R2 (95% CI)** |
| --- | --- |
| Age at diagnosis of type 2 diabetes | 28.68% (25.01% to 32.29%) |
| Sex | 0.073% (<0.01% to 0.092%) |
| Body mass index | 14.24% (11.02% to 17.65%) |
| Venous thromboembolism | 5.77% (0.22% to 10.55%) |
| Abdominal pain within previous 6 months | 16.45% (11.88% to 21.30%) |
| Weight loss within previous 6 months | 21.10% (14.71% to 27.70%) |
| Jaundice within previous 6 months | 40.46% (33.43% to 46.91%) |
| Heartburn within previous 6 months | 4.25% (0.05% to 10.81%) |
| Indigestion within previous 6 months | 5.03% (0.08% to 12.00%) |
| Nausea within previous 6 months | 13.41% (5.46% to 23.01%) |
| Prescribed use of digoxin | 0.61% (<0.01% to 3.9%) |
| HbA1c | 3.4% (0.17% to 5.57%) |
| ALT | 9.82% (6.99% to 12.96%) |
| Creatinine | 0.17% (<0.01% to 0.93) |
| Haemoglobin | 2.84% (1.32% to 4.88%) |
| Platelet count | 3.22% (1.58% to 5.35%) |

**Supplementary Table 9**. Predictive utility of individual predictors, as assessed using Royston & Sauerbrei’s R2. This was estimated by fitting a single-predictor Cox proportional hazards model on the full, final study cohort to provide a relative impression of the contribution of individual predictors.
